# *Arrabidaea chica* Hexanic Extract Induces Mitochondrion Damage and Peptidase Inhibition on *Leishmania* spp.

**DOI:** 10.1155/2014/985171

**Published:** 2014-04-09

**Authors:** Igor A. Rodrigues, Mariana M. B. Azevedo, Francisco C. M. Chaves, Celuta S. Alviano, Daniela S. Alviano, Alane B. Vermelho

**Affiliations:** ^1^Departamento de Produtos Naturais e Alimentos, Centro de Ciências da Saúde, Faculdade de Farmácia, Universidade Federal do Rio de Janeiro, 219491-590 Rio de Janeiro, RJ, Brazil; ^2^Programa de Pós Graduação Ciências dos Alimentos, Instituto de Química, Universidade Federal do Rio de Janeiro, 21941-590 Rio de Janeiro, RJ, Brazil; ^3^Embrapa Amazônia Ocidental, CP 319, 69010-970 Manaus, AM, Brazil; ^4^Departamento de Microbiologia Geral, Centro de Ciências da Saúde, Instituto de Microbiologia Paulo de Góes, Universidade Federal do Rio de Janeiro, 21941-590 Rio de Janeiro, RJ, Brazil

## Abstract

Currently available leishmaniasis treatments are limited due to severe side effects. *Arrabidaea chica* is a medicinal plant used in Brazil against several diseases. In this study, we investigated the effects of 5 fractions obtained from the crude hexanic extract of *A. chica* against *Leishmania amazonensis* and *L. infantum*, as well as on the interaction of these parasites with host cells. Promastigotes were treated with several concentrations of the fractions obtained from *A. chica* for determination of their minimum inhibitory concentration (MIC). In addition, the effect of the most active fraction (B2) on parasite's ultrastructure was analyzed by transmission electron microscopy. To evaluate the inhibitory activity of B2 fraction on *Leishmania* peptidases, parasites lysates were treated with the inhibitory and subinhibitory concentrations of the B2 fraction. The minimum inhibitory concentration of B2 fraction was 37.2 and 18.6 **μ**g/mL for *L. amazonensis* and *L. infantum*, respectively. Important ultrastructural alterations as mitochondrial swelling with loss of matrix content and the presence of vesicles inside this organelle were observed in treated parasites. Moreover, B2 fraction was able to completely inhibit the peptidase activity of promastigotes at pH 5.5. The results presented here further support the use of *A. chica* as an interesting source of antileishmanial agents.

## 1. Introduction

Among individual infectious diseases leishmaniasis is in the ninth position of the global burden of diseases. This illness has two main clinical manifestations which are cutaneous lesions (cutaneous leishmaniasis—CL) and visceral impairments (visceral leishmaniasis—VL) [1]. CL and VL represent a serious public health problem in 98 countries and 3 territories on 5 continents where the disease can be found. According to World Health Organization (WHO), there are more than 220,000 CL cases and 58,000 VL cases per year [2]. In Brazil, CL and VL are widespread and can be found not only in rural areas but also in urban areas mainly due to deforestation and new settlements [3].

Despite the large number of both synthetic and natural antileishmanial agents described in the literature, only a few drugs have reached the clinical stage with approval for human use. This fact could be partly explained by the lack of investments in drug research for poverty-related diseases, which includes leishmaniasis [4]. The current chemotherapy for leishmaniasis treatment still relies on the use of pentavalent antimonials and amphotericin B, although liposomal amphotericin B, paromomycin, and miltefosine have been introduced for the treatment of the disease in several countries. However, most of these drugs are expensive, present toxic effects, and are able to induce parasite resistance [5]. Consequently, the search for new and more effective antileishmanial agents remains crucial.

*Arrabidaea chica* (HBK) Verlot, Bignoniaceae, is a scrambling shrub native to tropical America, more particularly in the Amazon basin where it is also known as “Pariri,” “Crajirú,” “Carajuru,” or “Carajiru.” The leaves of* A. chica* have been traditionally used by Brazilian Indians as a dye for body painting in rituals and to protect the skin against sunlight as well as an insect repellant. Chemical investigations have been carried out since the beginning of this century to determine the composition of the* A. chica* dye, which used to be commercialized as such [6]. Nowadays* A. chica* is used by the regional population as an anti-inflammatory and astringent agent as well as a remedy for intestinal colic, diarrhea, leucorrhea, anemia, and leukemia [7]. The present study aimed to evaluate the antileishmanial effects of the hexanic extract of the* A. chica* leaves.

## 2. Materials and Methods

### 2.1. Chemicals

Resazurin, RPMI 1640 medium, and bovine serum albumin were purchased from Sigma Chemical Co., USA. Amphotericin B was purchased from Fontoura-Wyeth, Brazil. Fetal bovine serum (FBS) was purchased from Cripion Biotecnologia Ltda, Brazil. All solvents used were spectroscopic grade from Tedia (Fairfield, OH, USA). Column chromatographic product was purchased from Merck (Darmstadt, Germany).

### 2.2. Plant Material and Acquisition of the Hexanic Extract

The sample of* A. chica* was kept in a germplasm bank under the same cultivation practices at the EAFM Herbarium from Federal Institute of Amazonas (Manaus, AM), where a voucher specimen was deposited (registry EAFM 6791). Leaves of* A. chica* were collected between 08:00 and 09:00 AM.

*A. chica* crude extract was obtained by 1 week extraction in hexane. Then, the extract was carefully filtered, dried, and stored in opaque glass vials at −10°C. Afterwards, the crude extract was subjected to silica gel column chromatography with an increasing gradient of polarity, starting with 100%* n*-hexane and 100% ethyl acetate to 100% ethanol, affording five fractions (B1, B2, B3, B4, and B5).

### 2.3. Analysis of the Hexanic Extract of* A. chica* by GC-MS

The B2 fraction of the hexanic extract from “crajirú” was analyzed by a gas chromatograph (GC) interfaced to a mass spectrometer (MS) employing the following conditions: the oven temperature was programmed from 60°C to 300°C at 10°C/min, and helium was the carrier gas (at 1.0 mL/min). One microliter of 1% solution of the B2 fraction in dichloromethane was injected in split mode (1 : 50). Mass spectra were obtained in an Agilent 5973N system, fitted with a low bleeding 5% phenyl/95% methyl silicone (HP-5 MS, 30 m × 0.25 mm × 0.25 *μ*m) fused silica capillary column, operating in the electronic ionization mode (EI) at 70 eV, with a scan mass range of 40–500 m/z. Sampling rate was 3.15 scan/s. The ion source was kept at 230°C, mass analyzer at 150°C, and transfer line at 260°C. Linear retention indices (LRI) were measured by injection of a series of* n*-alkanes (C_10_–C_30_) in the same column and conditions as described above and compared with reference data. The identification of the B2 fraction constituents was made based on the retention indexes and by comparison of mass spectra with computer search using NIST21 and NIST107 libraries. Compound concentrations were calculated from the GC peak areas, and they were arranged in order of GC elution.

### 2.4. Parasite Strains and Cell Cultures

Promastigote forms of two* Leishmania* species,* Leishmania (L.) amazonensis* (IFLA/BR/1967/PH8) and* L. (L.) infantum* (MHOM/BR/1974/PP75) from the Leishmania Type Culture Collection of Oswaldo Cruz Institute/Fiocruz (Rio de Janeiro/RJ/Brazil) were used in all experiments. Parasites were axenically cultured in PBHIL medium as previously described [8]. In order to assure infectiveness of the parasites, periodical infection of mice peritoneal macrophages was performed.

### 2.5. Evaluation of* Leishmania* Inhibitory Concentrations

The assay was carried out in a 96-well microtiter plate where the hexanic extract from* A. chica* was serially diluted in duplicate to final test concentrations (1–500 *μ*g/mL). Then 5.0 × 10^5^ promastigote forms of* L. amazonensis* or* L. infantum* were harvested at the early stationary phase, added to each well, and plated at 26°C for 120 h. At the end of incubation period, 25 *μ*L of resazurin solution (5 mg/100 mL of phosphate buffer saline, pH 7.2) was added and the viability of parasites was determined in accordance with the protocol previously described [9]. The minimal inhibitory concentration (MIC) was considered the lowest concentration of the hexanic extract that completely prevented the growth of* Leishmania in vitro*. Alternatively, 120 h-treated parasites were centrifuged (1,000 g/5 min), washed twice in PBS, and then reincubated in fresh PBHIL culture medium in order to evaluate the leishmanicidal effect. The lowest concentration able to inhibit parasite growth was considered the minimal leishmanicidal concentration (MLC). The 50% inhibitory concentration (IC_50_) was determined by logarithmic regression analysis of the data obtained as described above.

### 2.6. Ultrastructure Analysis

Alterations in the ultrastructure of the parasites were analyzed by transmission (TEM) electron microscopy. First, promastigote forms of* L. infantum* were harvested at the early stationary phase of growth, washed twice with PBS, and incubated in the presence of a subinhibitory concentration (subMIC) of* A. chica* hexanic extract at 28°C for 24 hours. After the parasites were washed twice in PBS they were fixed with glutaraldehyde solution (2.5% glutaraldehyde in 0.1 M sodium cacodylate buffer containing 3.5% sucrose, pH 7.4) at 4°C for 60 min. Samples of treated cells and their controls (untreated cells) were sent to Plataforma Rudolf Barth (Instituto Oswaldo Cruz/Fiocruz/RJ) and processed as previously mentioned [10]. The photomicrographs were obtained using an electron microscope Jeol JEM1011.

### 2.7. Peptidase Inhibition Assay

*L. amazonensis* and* L. infantum* promastigotes (10^6^ parasites/mL) were harvested at the log phase, washed twice by centrifugation (1,500 ×g/5 min) with PBS pH 6.8, and then disrupted through seven cycles of freezing and thawing (−80°C/37°C). The cellular extracts were then centrifuged (12,000 ×g/10 min) and the supernatant aliquots preserved at 0°C. Peptidase (gelatinase) activity was analyzed through the protocol adapted from Cedrola et al. [11]. Briefly, 100 *μ*L of the cellular lysates was incubated with different concentrations of the hexanic extract in a PBS 0.1 M pH 5.5, or pH 10, and gelatin 1% mixture. E64 (cysteine peptidase inhibitor) and 1,10-phenanthroline (metalopeptidase inhibitor) were used as positive controls. After the 30 min incubation period at 37°C, enzymatic activity was stopped with isopropanol and the samples were refrigerated at 4°C for 15 min. Next, the samples were centrifuged (2,500 ×g/15 min) and 100 *μ*L supernatant was collected and the absorbance was measured as previously described [12]. One unit of gelatinase activity was defined as the amount of enzyme required to produce 1 *μ*g of peptides under the described assay conditions.

### 2.8. Peritoneal Mouse Macrophages and Cytotoxicity Assay

Nonelicited peritoneal macrophages from female Balb/c mice were collected in cold RPMI 1640 medium and plated in 96-well culture plates at the concentration of 10^5^ cells/100 *μ*L. Different concentrations, ranging from 1 to 500 *μ*g/mL, of the hexanic extract were added to each well and the cells were incubated at 37°C in 4% CO_2_ atmosphere for 48 h. The minimum cytotoxic concentration (MCC) was determined as previously described by Al-Musayeib et al. using resazurin as the cellular viability indicator [13]. The selective index (SI) was calculated using the MIC/MCC ratios. The animals used for macrophage acquisition were killed according to the federal guidelines and institutional policies by cervical dislocation.

### 2.9. Infection of Macrophages, Anti-Intracellular Amastigote Activity, and Nitric Oxide Production

Peritoneal mouse macrophages were obtained as described above. The infection assays were carried out following the protocol described by Passero et al. with slight modifications [14]. Briefly, peritoneal macrophages (10^5^ cells/100 *μ*L) were plated in 96-well culture plates and a ratio of 5 stationary phase promastigotes (*L. amazonensis* or* L. infantum*) to 1 macrophage was used for the infection procedure. The parasite-macrophage interactions were carried out in RPMI 1640 medium supplemented with 10% of FBS at 35°C for 24 hours in 4% CO_2_ atmosphere. After interaction assays were completed free promastigotes and nonadherent macrophages were removed by extensive washing with PBS and the hexanic extract was added to each well at inhibitory and subinhibitory concentrations for* L. amazonensis* and* L. infantum*. After 48 hours of treatment the supernatants from* L. amazonensis*- and* L. infantum*-infected macrophages were analyzed for their nitrite contents by Griess reaction [15]. Then the plates were washed four times with PBS and cultures were incubated in PBHIL medium supplemented with 10% of FBS for 72 hours at 26°C to evaluate the number of promastigote forms differentiated into the medium. The number of viable promastigotes was determined using a hemocytometer chamber.

## 3. Results and Discussion

In the present study we investigated the antileishmanial effects of the hexanic extract from* A. chica* against two* Leishmania* species, the causative agents of cutaneous and visceral leishmaniasis,* L. amazonensis* and* L. infantum*, respectively.  Table 1 summarizes the inhibitory activity of five fractions obtained from the crude hexanic extract on the growth of the parasites tested. B2 (1 : 1* n*-hexane/ethyl acetate) was the most active fraction with MIC values of 37.2 and 18.6 *μ*g/mL for* L. amazonensis *and* L. infantum *promastigotes, respectively. Recently, the antimicrobial activity of a hydroethanolic extract from* A. chica* was reported against* Helicobacter pylori* and* Enterococcus faecalis* demonstrating the potential of this plant as a source of biologically active molecules [16]. Only a few species from the* Arrabidaea* genus have been investigated for their antiprotozoal activity. In a study conducted by Barbosa et al. the ethanol extract from* A. chica* and fractions were active against* Trypanosoma cruzi* trypomastigotes, but high concentrations were needed to cause parasite lyses (4.0 and 2.0 mg/mL, resp.) [6]. Triterpenoids isolated from an* A. triplinervia* ethanol extract have been shown to present anti-*T. cruzi* activity [17]. However, the crude ethanol extract as well as the isolated compounds, ursolic acid and oleanolic acid, caused* in vitro* elimination of trypomastigotes at high concentrations of 5.0, 0.4, and 1.6 mg/mL, respectively. In the present study, the reincubation of parasites treated at MIC values in fresh medium revealed that those cells were no longer able to grow. Thus, the inhibitory activity observed was leishmanicidal for the promastigote forms of* L. amazonensis* and* L. infantum* (MIC values = MLC values).

**Figure 1:**
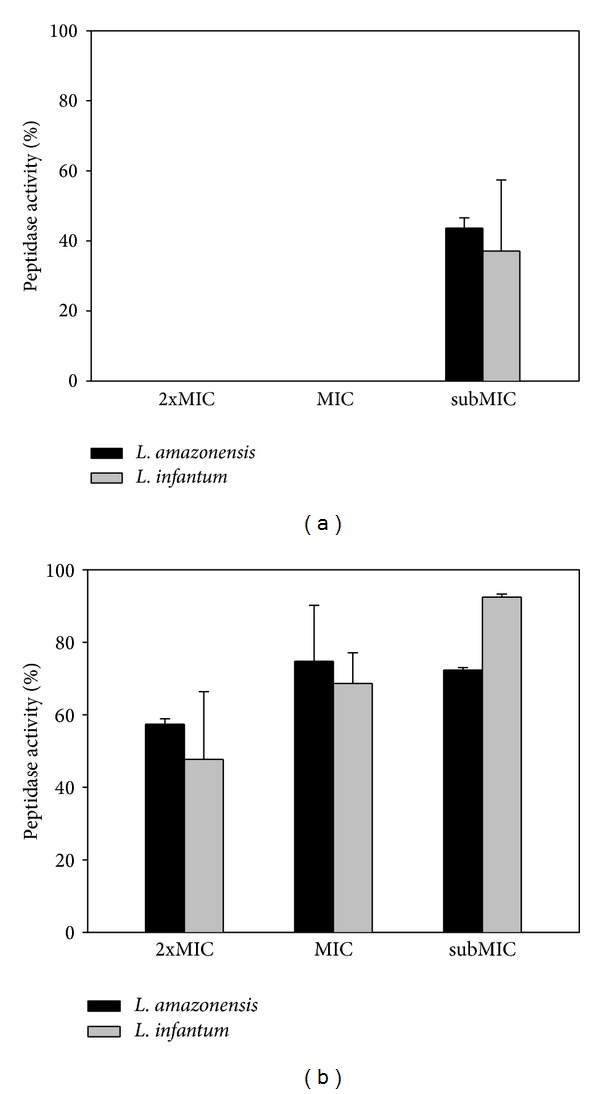
Inhibitory activity of the B2 fraction obtained from the* A. chica* hexanic extract on* Leishmania* peptidases. (a) Peptidase inhibitory activity at pH 5.5; (b) peptidase inhibitory activity at pH 10. 2xMIC, MIC, and subMIC values for* L. amazonensis* and* L. infantum* were 74.4, 37.2, and 18.6 *μ*g/mL and 37.2, 18.6, and 9.3 *μ*g/mL, respectively.

*Leishmania* promastigotes were shown to be more sensitive to B2, and therefore the chemical analysis of this fraction was carried out and the main components identified were linolenic acid, methyl ester (25.38%)* n*-hexadecanoic acid (19.61%), octadecanoic acid (14.10%), and gamma-sitosterol (12.85%) as shown in Table 2. Fatty acids have been reported to be active against* Leishmania*; however the activity of such compounds seems to be related mainly to unsaturated fatty acids rather than their saturated analogues [18]. The fatty acid-rich methanol extract from* Ulva lactuca* displayed antitrypanosomal and antileishmanial activities through parasite motility inhibition at low concentrations (<100 *μ*g/mL). In that work,* n*-decanoic acid,* n*-dodecanoic acid, and* n*-hexadecanoic acid were described as the main active compounds [19]. More recently, the action mechanism of some fatty acids has been related to topoisomerase IB inhibition as demonstrated by Carballeira et al., during the evaluation of the antileishmanial activity of *α*-methoxylated fatty acids [20]. Here, the B2 fraction demonstrated significant peptidase inhibition when tested on cellular lysates of* Leishmania* (Figure 1). After incubation of the cellular lysates treated at MIC (37.2 and 18.6 *μ*g/mL) and twice MIC (74.4 and 37.2 *μ*g/mL) of B2 with phosphate buffer 5.5 pH at 37°C, peptidase activity was completely inhibited for* L. amazonensis* and* L. infantum*, respectively (Figure 1(a)). E64 (cysteine peptidase inhibitor) was used as the positive control completely inhibiting peptidase activity when incubated with* Leishmania* lysates under the same conditions. In order to evaluate the effect of the B2 fraction on metalopeptidases, cellular lysates from* L. amazonensis* and* L. infantum* were also incubated with phosphate buffer 10.0 pH (Figure 1(b)). Despite the decrease in peptidase activity the results show that B2 was less effective against those peptidases.

**Figure 2:**
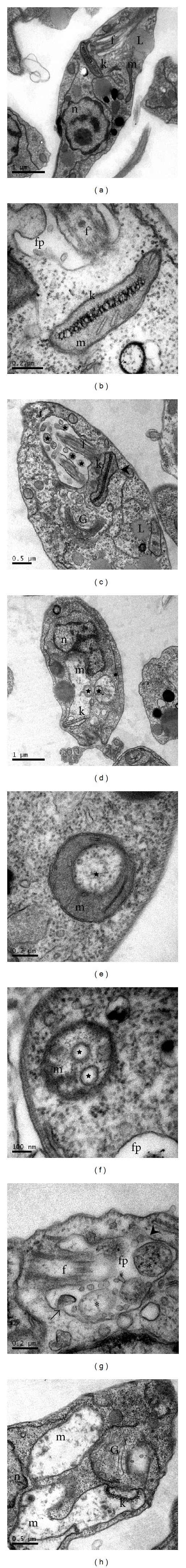
Transmission electron microscopy of* L. infantum* promastigotes treated with the B2 fraction from the* A. chica* hexanic extract. ((a)-(b)) Thin sections of untreated promastigote forms displaying normal morphology and intracellular structures. (b) Detail of the mitochondrion containing the kinetoplast (k). ((c)–(h)) Parasites treated for 24 hours with subMIC (9.3 *μ*g/mL) or MIC (18.6 *μ*g/mL) of the B2 fraction, showing serious cellular damage. (c) Parasite treated with subMIC value of the B2 fraction presenting a dilated flagellar pocket with the presence of several vacuoles (∗). Note the rupture of the mitochondrion membrane (arrowhead in (c)). (d) Parasite treated with the B2 fraction at MIC value (18.6 *μ*g/mL) presenting mitochondrial swelling and some vesicles (★) inside this organelle; (details of intramitochondrial vesicles in (e) and (f)); (g) in detail, parasite flagellar pocket showing intense release of vesicles with cytoplasmic content (∗); (h) in detail, increased mitochondrial volume and Golgi complex alterations. n, nucleous; m, mitochondrion; k, kinetoplast; f, flagellum; fp, flagellar pocket; G, Golgi complex; L, lipid.

Naturally occurring sterols also present antileishmanial activity. In a study conducted by Pan et al. sterols obtained from the roots of* Pentalinon andrieuxii* displayed the best inhibitory activities (IC_50_) at concentrations ranging from 9.2 to 30.0 *μ*M and 0.03 to 3.5 *μ*M against promastigote and amastigote forms of* L. mexicana*, respectively [21]. The authors attributed the antileishmanial activity of the sterols tested to membrane alterations caused by cholesterol replacement during its biosynthesis. In the present study, important alterations on the ultrastructure of* L. infantum* promastigotes were also observed on B2-treated parasites (Figure 2). Parasites presented abnormal cell body shapes after 24 h exposure to the B2 fraction at 18.6 *μ*g/mL (MIC), when compared to untreated parasites (Figure 2(b)). Mitochondrial dilatation with loss of matrix contents and Golgi complex alterations followed by a cytoplasm vacuolization process were also observed (Figure 2(c)). In addition, an intense exocytic process of cytoplasmic content into the flagellar pocket was noted. Mitochondrion seems to be a common target for several natural products, crude extracts, or isolated compounds, as has been reported by several researchers [10, 22–24]. The mode of action of most natural products that cause mitochondrial damage and parasite death has been attributed mainly to sterol biosynthesis inhibition and mitochondrial membrane potential dysfunction [25–27]. Here, the* L. infantum* mitochondrion was drastically damaged by the B2 fraction obtained from* A. chica* hexanic extract as shown in Figure 2. An intense swelling of the mitochondrion with the presence of vesicles was observed in the parasites. In some cases, the mitochondrion membrane appears to be disrupted.

**Figure 3:**
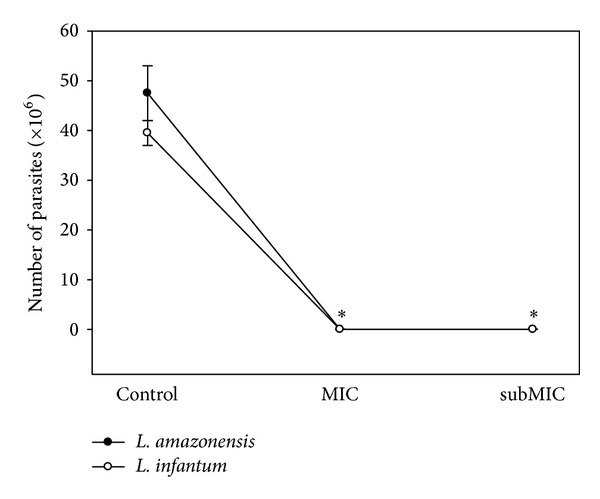
Anti-intracellular amastigote activity of the B2 fraction from the* A. chica* hexanic extract. B2-treated macrophages previously infected with* Leishmania* were incubated in fresh medium at 28°C for 72 hours. The number of promastigote forms obtained from macrophages cultures was counted using a hemocytometer chamber. Each point represents the mean ± S.E. of 2 independent experiments performed in triplicate. MIC and subMIC values for* L. amazonensis* and* L. infantum* were 37.2 and 18.6 *μ*g/mL and 18.6 and 9.3 *μ*g/mL, respectively. Asterisks indicate that treated parasites were statistically different (*P* < 0.05) from control parasites.

**Figure 4:**
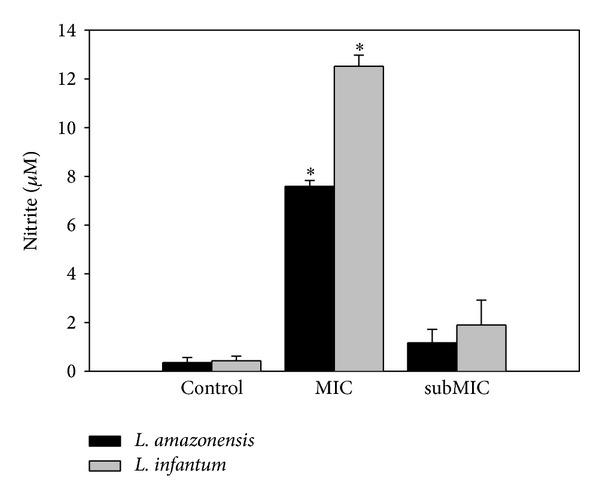
Nitric oxide synthesis by* Leishmania*-infected macrophages treated with the B2 fraction. After 48 hours treatment with the B2 fraction the supernatant from* L. amazonensis*- and* L. infantum*-infected macrophages was collected and the nitrite content determined through Griess reaction. Each point represents the mean ± S.E. of 2 independent experiments performed in triplicate. MIC and subMIC values for* L. amazonensis* and* L. infantum* were 37.2 and 18.6 *μ*g/mL and 18.6 and 9.3 *μ*g/mL, respectively. Asterisks indicate that treated parasites were statistically different (*P* < 0.05) from control parasites.

The B2 fraction from the* A. chica* hexanic extract demonstrated antileishmanial activity during the infection of peritoneal mice macrophages. After a 48-hour treatment with B2 at MIC and subMIC values, the number of promastigotes recovered in the supernatants of* L. amazonensis*- and* L. infantum*-infected macrophage cultures was drastically reduced when compared to controls (Figure 3). According to Passero et al. only viable amastigotes are able to differentiate to promastigotes under established conditions [14]. Besides, after treatment with 37.2 and 18.6 *μ*g/mL of the B2 fraction, the nitrite contents detected in the supernatant of* L. amazonensis*-infected macrophages was higher than those found on untreated cells cultures, about 7.6 and 1.16 *μ*M, respectively. With the* L. infantum* model of infection, the nitrite content detected on MIC and subMIC-treated cultures was about 12.5 and 2.0 *μ*M, respectively (Figure 4). These results give additional evidence of the enhancement of macrophage killing mechanism elicited by* A. chica* against the intracellular form of* Leishmania*. Moreover,* A. chica* has been described as a potent wound healing agent able to stimulate fibroblast growth and collagen synthesis at 30 *μ*g/mL (EC_50_) and 250 *μ*g/mL, respectively.* In vivo* assays demonstrated an impressive reduction of lesion size of about 96% [28]. In addition, Lima de Medeiros et al. reported the hepatoprotective activity of the hydroethanolic extract of* A. chica* based on the suppression of hepatic markers such as serum glutamic oxaloacetate transaminase (GOT) and glutamic pyruvate transaminase (GPT) and decreasing levels of plasma bilirubin [29]. Considering the clinical manifestations related to cutaneous and visceral leishmaniasis, such as skin lesions and hepatic damage, respectively,* A. chica* could represent a promising phytotherapeutic agent.

In conclusion, the hexanic extract from* A. chica*, especially the B2 fraction, possesses activity against* L. amazonensis* and* L. infantum*. Fatty acids and sterols probably are the main components involved in the antileishmanial activity, but further investigation will be necessary in order to evaluate the isolated compounds. Taken together, the results presented herein in addition to those reported in literature concerning tissue healing effects and liver protection of* A. chica* are motivation for further investigation of this plant using* in vivo* models of* Leishmania* spp. infection.

## Figures and Tables

**Table 1:** Antileishmanial activity and cytotoxic effect of the *A. chica* hexanic extract fractions.

Hexanic extract fraction	*L. amazonensis *		*L. infantum *		Macrophages	SI
	MIC (*μ*g/mL)	IC_50 _(*μ*g/mL)	MIC (*μ*g/mL)	IC_50 _(*μ*g/mL)	MCC (*μ*g/mL)	
B1	na	nd	na	nd	nd	nd
B2	37.2	31.8	18.6	14.7	297.6	8.0^a^/16^b^
B3	186.7	152.2	186.7	139.6	nd	nd
B4	368	198.5	368	179.7	nd	nd
B5	na	nd	na	nd	nd	nd
Amphotericin B	1.01	0.07	0.625	0.01	14.6	14.4^a^/23.4^b^

MIC: minimum inhibitory concentration; IC_50_: 50% inhibitory concentration; MCC: minimum cytotoxic concentration.

na: not active at the highest concentration tested (500 *μ*g/mL); nd: not determined.

SI: selective index; ^a^selective index for *L. amazonensis*; ^b^selective index for *L. infantum*.

**Table 2:** Chemical characterization of the B2 fraction obtained from the *A. chica *hexanic extract.

RT	LRI	Components	%
15.876	1968	*n*-Hexadecanoic acid	19.61
17.294	2114	Phytol	3.05
17.508	2143	Linoleic acid	6.36
17.575	2150	Linolenic acid, methyl ester	25.38
17.777	2173	Octadecanoic acid	14.10
19.517	2363	Eicosanoic acid	1.31
25.290	3031	Vitamin E	4.94
26.244	3176	Campesterol	1.60
26.491	3206	Stigmasterol	4.02
27.135	3417	Gamma-sitosterol	12.85

% total			93.22

RT: retention time.

LRI: linear retention indices.

## References

[1] Barrett MP, Croft SL (2012). Management of trypanosomiasis and leishmaniasis. *British Medical Bulletin*.

[2] WHO Leishmaniasis: epidemiology and access to medicines—an update based on the outcomes of WHO regional meetings, literature review and experts' opinion.

[3] Alvar J, Vélez ID, Bern C (2012). Leishmaniasis worldwide and global estimates of its incidence. *PLoS ONE*.

[4] Ehrenberg JP, Ault SK (2005). Neglected diseases of neglected populations: thinking to reshape the determinants of health in Latin America and the Caribbean. *BMC Public Health*.

[5] den Boer M, Argaw D, Jannin J, Alvar J (2011). Leishmaniasis impact and treatment access. *Clinical Microbiology and Infection*.

[6] Barbosa WLR, Pinto LDN, Quignard E, Vieira JMDS, Silva JOC, Albuquerque S (2008). *Arrabidaea chica* (HBK) Verlot: phytochemical approach, antifungal and trypanocidal activities. *Revista Brasileira de Farmacognosia*.

[7] Siraichi JTG, Felipe DF, Brambilla LZS (2013). Antioxidant capacity of the leaf extract obtained from *Arrabidaea chica* cultivated in Southern Brazil. *PLoS ONE*.

[8] Rodrigues IA, da Silva BA, dos Santos ALS, Vermelho AB, Alviano CS, do Socorro Santos Rosa M (2010). A new experimental culture medium for cultivation of *Leishmania amazonensis*: its efficacy for the continuous in vitro growth and differentiation of infective promastigote forms. *Parasitology Research*.

[9] Rolón M, Vega C, Escario JA, Gómez-Barrio A (2006). Development of resazurin microtiter assay for drug sensibility testing of *Trypanosoma cruzi* epimastigotes. *Parasitology Research*.

[10] Rodrigues IA, Azevedo MM, Chaves FC (2013). In vitro cytocidal effects of the essential oil from *Croton cajucara* (red sacaca) and its major constituent 7-hydroxycalamenene against *Leishmania chagasi*. *BMC Complementary and Alternative Medicine*.

[11] Cedrola SML, de Melo ACN, Mazotto AM (2012). Keratinases and sulfide from *Bacillus subtilis* SLC to recycle feather waste. *World Journal of Microbiology and Biotechnology*.

[12] Lowry OH, Rosebrough NJ, Farr AL, Randall RJ (1951). Protein measurement with the Folin phenol reagent. *The Journal of Biological Chemistry*.

[13] Al-Musayeib NM, Mothana RA, Matheeussen A, Cos P, Maes L (2012). In vitro antiplasmodial, antileishmanial and antitrypanosomal activities of selected medicinal plants used in the traditional Arabian Peninsular region. *BMC Complementary and Alternative Medicine*.

[14] Passero LFD, Bonfim-Melo A, Corbett CEP (2011). Anti-leishmanial effects of purified compounds from aerial parts of *Baccharis uncinella* C. DC. (Asteraceae). *Parasitology Research*.

[15] Green SJ, Meltzer MS, Hibbs JB, Nacy CA (1990). Activated macrophages destroy intracellular *Leishmania major* amastigotes by an L-arginine-dependent killing mechanism. *Journal of Immunology*.

[16] Mafioleti L, da Silva Junior IF, Colodel EM, Flach A, Martins DT (2013). Evaluation of the toxicity and antimicrobial activity of hydroethanolic extract of *Arrabidaea chica* (Humb. & Bonpl.) B. Verl. *Journal of Ethnopharmacology*.

[17] Leite JPV, Oliveira AB, Lombardi JA, Filho JDS, Chiari E (2006). Trypanocidal activity of triterpenes from *Arrabidaea triplinervia* and derivatives. *Biological and Pharmaceutical Bulletin*.

[18] Carballeira NM, Cartagena MM, Prada CF, Rubio CF, Balaña-Fouce R (2009). Total synthesis and antileishmanial activity of the natural occurring acetylenic fatty acids 6-heptadecynoic acid and 6-icosynoic acid. *Lipids*.

[19] Cunningham LV, Kazan BH, Kuwahara SS (1972). Effect of long-chain fatty acids on some trypanosomatid flagellates. *Journal of General Microbiology*.

[20] Carballeira NM, Cartagena M, Li F (2012). First total synthesis of the (±)-2-methoxy-6-heptadecynoic acid and related 2-methoxylated analogs as effective inhibitors of the leishmania topoisomerase IB enzyme. *Pure and Applied Chemistry*.

[21] Pan L, Lezama-Davila CM, Isaac-Marquez AP (2012). Sterols with antileishmanial activity isolated from the roots of *Pentalinon andrieuxii*. *Phytochemistry*.

[22] Rosa MDSS, Mendonça-Filho RR, Bizzo HR (2003). Antileishmanial activity of a linalool-rich essential oil from *Croton cajucara*. *Antimicrobial Agents and Chemotherapy*.

[23] Sen N, Das BB, Ganguly A (2004). Camptothecin induced mitochondrial dysfunction leading to programmed cell death in unicellular hemoflagellate *Leishmania donovani*. *Cell Death and Differentiation*.

[24] Brenzan MA, Santos AO, Nakamura CV (2012). Effects of (-) mammea A/BB isolated from *Calophyllum brasiliense* leaves and derivatives on mitochondrial membrane of *Leishmania amazonensis*. *Phytomedicine*.

[25] Fonseca-Silva F, Inacio JDF, Canto-Cavalheiro MM, Almeida-Amaral EE (2011). Reactive oxygen species production and mitochondrial dysfunction contribute to quercetin induced death in *Leishmania amazonensis*. *PLoS ONE*.

[26] Medina JM, Rodrigues JC, de Souza W, Atella GC, Barrabin H (2012). Tomatidine promotes the inhibition of 24-alkylated sterol biosynthesis and mitochondrial dysfunction in *Leishmania amazonensis* promastigotes. *Parasitology*.

[27] Monzote L, García M, Pastor J (2014). Essential oil from *Chenopodium ambrosioides* and main components: activity against *Leishmania*, their mitochondria and other microorganisms. *Experimental Parasitology*.

[28] Jorge MP, Madjarof C, Gois Ruiz AL (2008). Evaluation of wound healing properties of *Arrabidaea chica* Verlot extract. *Journal of Ethnopharmacology*.

[29] Lima de Medeiros B, dos Santos Costa K, Alves Ribeiro JF, Carrera Silva JO, Ramos Barbosa WL, Tavares Carvalho JC (2011). Liver protective activity of a hydroethanolic extract of *Arrabidaea chica* (Humb. and Bonpl.) B. Verl. (pariri). *Pharmacognosy Research*.

